# Laser Deposited 18Ni300 Alloy Powder on 1045 Steel: Effect of Passes and Preheating on Microstructure

**DOI:** 10.3390/ma15031209

**Published:** 2022-02-05

**Authors:** Omid Emadinia, Jorge Gil, Rui Amaral, Cláudia Lopes, Rui Rocha, Ana Reis

**Affiliations:** 1 LAETA/INEGI—Institute of Science and Innovation in Mechanical and Industrial Engineering, 4200-465 Porto, Portugal; jgil@inegi.up.pt (J.G.); ramaral@inegi.up.pt (R.A.); areis@inegi.up.pt (A.R.); 2 Faculty of Engineering, University of Porto, 4200-465 Porto, Portugal; ctlopes@fe.up.pt; 3 CEMUP, Materials Centre of the University of Porto, Rua do Campo Alegre, 823, 4150-180 Porto, Portugal; lmev@cemup.up.pt

**Keywords:** maraging 18Ni300, direct energy deposition, microstructure, microhardness, heat affected zone, dilution, grains and crystallographic information

## Abstract

The application of maraging steels such as 18Ni300 alloy is noteworthy for mould industries, applying repair purposes through direct energy deposition process. This objective requires microstructural characterizations and the evaluation of mechanical behaviour such as hardness. The state of substrate material, including the heat-affected zone (HAZ) and the interface between the HAZ and deposited layer, is essential, the formation of hard phases and abrupt transitions. Thus, the influence of the number of deposited layers or the pre-heating condition appears noteworthy. In the current study, microscopy observations did not reveal the presence of any crack in the cross-sections of deposited 18Ni300 alloy powder on AISI 1045 sheet steel; however, pores were observed in deposited layers. Besides, microscopic analyses revealed the achievement of a smooth HAZ in the deposited layers composed of three-layered depositions or that received preheating, confirmed by hardness measurements as well. Dilution effect ensured a metallurgical bonding between depositions and substrate, strongly affected by preheating. The HAZ microstructure, mainly martensitic transformation, distribution of chemical composition, epitaxial growth at the interface, and the size of crystals and grains were affected by preheating or the number of layers. Moreover, the heat propagation and/or dissipation across the deposited layers influenced the dendrite morphology and the texture of grains. The preheating condition provoked the formation of cellular/equiaxed dendrites that was highlighted in the three-layered deposition, increase in dendrite interspace growth.

## 1. Introduction

Direct Energy Deposition (DED), initially a process focused on surface cladding, has recently appeared as a valid additive manufacturing (AM) process in which feedstock material (either in powder or wire form) is melted as it is ejected or introduced into the melting pool. A feeder system assures the delivery of the feedstock to the melting pool generated by an adequate heat source, and an inert gas protects the melting pool. The heat source may vary, but it usually is a fibre or a solid-state laser [1]. DED process presents several advantages: cladding surfaces over substrates with complex geometries, as it does not require a powder bed [2]; producing large components or reaching to specific zones of a component through a robotic arm; manufacturing components composed of functionally graded materials (FGMs) using powder feedstocks, these are advanced materials whose chemical and/or metallurgical configuration gradually changes along the building direction; displaying greater productivity rates in comparison with other metallic additive manufacturing (MAM) techniques [3]; however, DED cannot produce surface finishing as good as that of produced by powder bed fusion methodologies [4]. DED processing parameters involve the laser power, the scanning speed, which is defined as the speed at which the heat source travels with respect to the substrate, and the powder feeding rate, defined by the rate at which feedstock is introduced into the melting pool. Regarding the influence of the laser power, some authors concluded that it is the predominant parameter in the quantity of melted substrate, especially when the feeding rates are low (below 20 g/min for a laser spot size of 3 mm) [5]. Recent studies related the laser power and powder feed rate with the development of defects for a repairing procedure of a 630 stainless steel sample, concluding that a reduction in powder feed rate for a constant power output proved the effectiveness in the reduction in defects in the interface between the deposited material and the substrate [6]. The scanning speed effect on microstructural evolution was the subject of a recent work, in which several scanning speeds were employed during the deposition of a 12CrNi2 alloy, highlighting a decrease in the grain size by increasing scanning speed [7]. Other relevant parameters should be further considered, such as the employment of substrate pre-heating, shielding gas flow rate, the carrier gas flow rate, component scanning pattern, nozzle-to-substrate distance, laser spot size, laser wavelength and laser energy profile [1].

One of the main applications of DED lies in repairing components with successful performance. Several authors [8,9,10] used DED-based technologies to repair damaged turbine blades: in one case, the original shape of the Nistelle 625 turbine blade was restored with a mean accuracy of 0.030 mm, maintaining the strength and ductility of the original material [9]; alternatively, a turbine blade repairing operation was successfully repaired using an alloy comprised of 25% Merl 72 and 75% Rene 142, in which the deposited portion showed superior mechanical properties [10]. DED systems are also associated with the remanufacturing of mould dies, which are usually produced costly, highlighting a great financial incentive to extend their life cycle [11]: some authors revealed a successful repair of dies applied in the automotive industry, where the repaired component had a life time similar to the original part [12]; AISI H11 tool steel dies were repaired, highlighting the importance of minimizing the mismatch between the metallurgical configuration of the repaired zone and the surrounding material [13]. Additionally, AISI H13 tool steel substrates were repaired with the same material by DED; the microstructure, hardness and wear resistance of the substrate did not change after deposition, the deposited alloy was hard, 671 HV, attributed to microstructure affected by fast cooling rates during deposition [14].

Maraging steel is one of the most frequent alloys used in the mould industry, mainly for repair purposes [15]. This alloy is composed of a large concentration of nickel, medium levels in cobalt and molybdenum, a small amount in titanium and a very low carbon concentration, i.e., the Ni (for 18–25 weight %) leads to reduce the start temperature of martensite transformation, Ms, to 150 °C, while austenite is not formed upon reheating until temperatures above 500 °C; this characteristic allows the alloy to be subjected to temperatures above 400 °C, leading to the precipitation of intermetallic phases which contribute to strengthening. The Mo and Ti precipitate as Ni3Mo, Ni3Ti and Laves; the Co reduces the solubility of Mo, increasing in precipitation [16]. The low C concentration, which is smaller than 0.03%, is responsible for avoiding the precipitation of titanium carbide (TiC); the increase in TiC precipitates deteriorates the impact strength and toughness [17]. The application of maraging steels are in the automotive, nuclear, and aerospace industries for having: high weldability and printability, withstanding thermal cycles associated with manufacturing processes as an alloy with considerable toughness; good machinability, especially before undergoing heat treatment, while at the same time boasting an average thermal expansion coefficient [18], this property allows subtractive machining and finishing operations to be performed before age hardening; having a combination of both high strength and toughness. 

The processing of maraging steels by MAM methodologies included powder-based technologies such as selective laser melting (SLM). Some authors [19] performed the SLM technique starting the optimization of printing parameters and then evaluating the influence of heat treatment. These authors achieved a maximum relative density of 99.8% using a scanning speed of 700 mm/s, laser power of 300 W and overlapping rate of 40%. Other researchers studied the interface between SLM-produced maraging and H13 tool steel, confirming a lath martensite formation across the interface between the substrate and cladding surface, the hardness profile appeared homogeneous after solution treatment; however, the non-heat treated specimen displayed a fined morphology with the growth direction collinear to the building direction [20]. A recent study evaluated the performance of constant levels of energy in SLM-produced 18Ni300 alloy, the as-built optimised alloy showed a cellular morphology [21]. 

Maraging steels were also produced by the DED technique, the deposition of this alloy on AISI 304L stainless steel alloy was optimised, observing an increase for at least 170 HV in the heat-treated deposition, highlighting the production of almost 100% dense deposition by the application of energy densities over 180 J/mm^2^ [22]. A recent study highlighted the significance of laser power on dilution, whereas the laser speed was very effective in the height, width, depth and porosity of deposited line beads [15]. Besides, a recent study revealed that the effectiveness of applying a deposition-pause strategy forming in-situ nano-precipitation led to an increase in hardness and yield strength, encouraging the possibility of eliminating postprocessing ageing treatment [23]. 

In the current study, the deposition of the maraging 18Ni300 alloy on a sheet steel of AISI 1045 alloy was performed, applying DED process. The powders are melted and incorporated with the material beneath, e.g., substrate or the previous layer. This structural steel is prone to martensitic transformation, as it contains a carbon concentration of almost 0.4–0.5%. Thus, the elimination of martensite will be noteworthy to avoid stress concentration. Thus, the performance of pre-heating substrate alloy for deposition was performed and evaluated. Moreover, the effect of the number of deposited layers was studies as well. The interface of the depositions with the substrate alloy and the depositions were analysed considering microstructure and hardness evolutions. Optical microscopy, scanning electron microscopy, energy dispersive X-ray spectroscopy, electron backscatter diffraction techniques were performed for microstructural, chemical and crystallographic analyses. The Vickers microhardness was used to characterize the topmost region of the deposition to the substrate, including the heat-affected zone. 

## 2. Materials and Methods

### 2.1. Powder and Substrate

**Figure 1:**
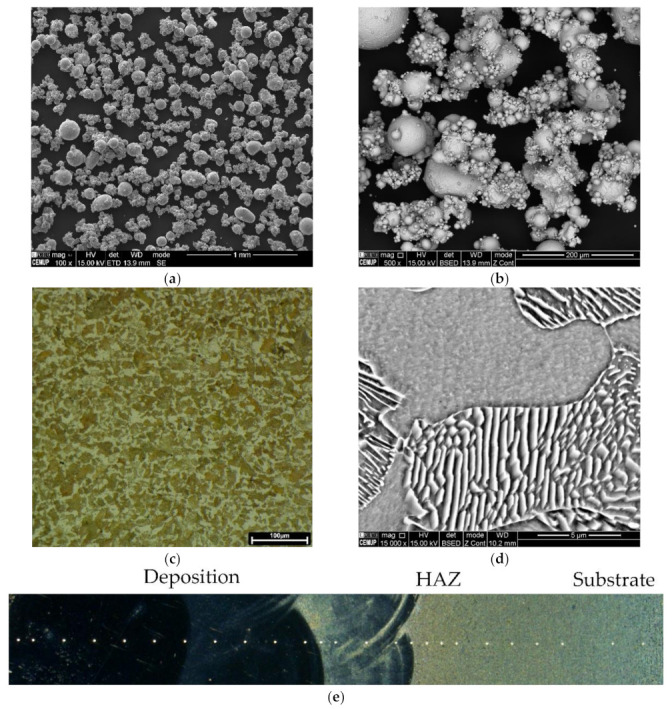
(**a**,**b**) The SEM image showing the morphology of 18Ni300 alloy powder particles used in the current study; (**c**,**d**) The microstructure of the 1045 steel, respectively, a digital OM image and a SEM image illustrating pearlite and ferrite grains in the virgin material; (**e**) a schematic illustrating the positioning of hardness indentations across the deposition towards HAZ and subtrate.

The W722 powder was supplied from Böhler Edelstahl GMBH, Germany. This powder is equivalent to the maraging 18Ni300 steel composed of C < 0.03, Si < 0.1, Mn < 0.15, P and S < 0.01, Cr < 0.25, 4.5 < Mo < 5.2, 17 < Ni < 19, 0.8 < Ti < 1.2, 8.5 < Co < 10 [24]. Figure 1a,b shows the powder particles used in the current study, fine to large spherical particles with a satellite-like morphology. As observed by SEM backscattered image mode in Figure 1b, the powder chemical composition appears to be homogeneous. Moreover, the particle size distribution of this powder is comprised of D10 of 48.8 μm, D50 of 89.6 μm, and D90 of 143.7 μm, analysed by dynamic light scattering methodology using a Coulter LS230 particle size analyser equipment.

The substrate material is AISI 1045 steel, rectified sheets of 23 mm thickness. 

### 2.2. Preheating Condition

The preheating of substrate was performed for some experiments in order to study the phase evolutions in the heat-affected zone (HAZ) and at the interface as well. The heating process was performed by a DEMMLER (Memmingen, Germany) 3D welding table fitted with eight heating cells distributed across the DED working platform; each cell was connected to a thermocouple, permitting temperature monitoring through a control interface outside of the work chamber. Even so, further temperature control was carried out by an RS52 (RS Pro, Madrid, Spain) digital thermometer, placed on the top surface of the substrate and measured before and after each deposition. Preheating temperature applied for this study was 400 °C, providing a cooling rate long enough to avoid martensitic transformation in the substrate during the deposition process and the cooling as well. The martensite start temperature (M_S_) of AISI 1045 steel appears to be around 350 °C to 380 °C [25].

### 2.3. Powder Deposition by Direct Energy Deposition

The laser deposition process in the current study was performed using Coherent Highlight FL3000 equipment, which is a fibre laser with a continuous wave (CW) mode, having a wavelength of 1070 nm ± 10 nm providing a maximum power of 3000 W. This equipment is connected to a double Medicoat AG Disk feeder, delivering to a Fraunhofer powder splitter, in turn diverting the powder and gas flow into four orifices of a COAX12V6 nozzle head with a capacity of temperature regulation up to 6000 W. The robotic arm is a 6-axis KUKA industrial robot. The Argon serves as the carrying and protecting gas during deposition, fed at a pressure of 6 bar. The employed parameters for producing depositions were based on a previous study [22], indicating that the application of larger scanning speeds and lower laser powers lead to a decrease in dilution. Thus, the ideal energy density for the present work was set at 60 J/mm^2^, requiring a laser power of 1850 W, scanning speed of 12 mm/s and feeding rate of 12 g/min. These laser power and scanning speed values were obtained considering the laser spot diameter of 2.5 mm.

In the current study, four conditions were produced: two individual depositions of 25 × 25 mm respectively without and with the preheating substrate, each composed of one-layered deposition; the two other depositions were produced similarly, each composed of three layers.

### 2.4. Characterization

Afterwards, transversal cross-sections, perpendicular to deposition direction, were prepared by conventional metallographic techniques for microstructural characterization, followed by a chemical etching to attach polished surfaces was 2% Nital solution. Metallography observations were performed using a digital optical microscope (OM) Leica DVM6 A, using LAS X version 3.014 software. Microscopy analyses such as local/profile/map chemical analyses and crystallographic evaluations were conducted using a scanning electron microscope (SEM), an FEI Quanta 400 FEG (ESEM, Hillsboro, OR, USA) equipment, using secondary electron (SE) or backscattered electron (BSE) modes. These analyses were respectively executed using energy-dispersive X-ray spectroscopy (EDS) (EDAX Genesis X4M, Oxford Instrument, Oxfordshire, UK), and electron backscatter diffraction (EBSD) (EDAX-TSL OIM EBSD, Mahwah, NJ, USA) technique using TSL OIM Analysis 5.2 software for creating inverse pole figure (IPF) maps or texture identification applying pole figure (PF) maps. Regarding EBSD, a clean-up routine was performed with a grain tolerance angle of 15°.

The hardness measurements were performed on these metallographic specimens, making three indentation lines on each specimen from the topmost region of the deposited area to the end of the HAZ reaching the substrate material. This mechanical behaviour was conducted using a ZwickRoell & Emco-Test DuraScan 10/20 G5 equipment, applying 100 gf indentation (HV 0.1).

## 3. Results and Discussion

### 3.1. Macrostructure Evaluations in Deposited Layers

**Figure 2:**
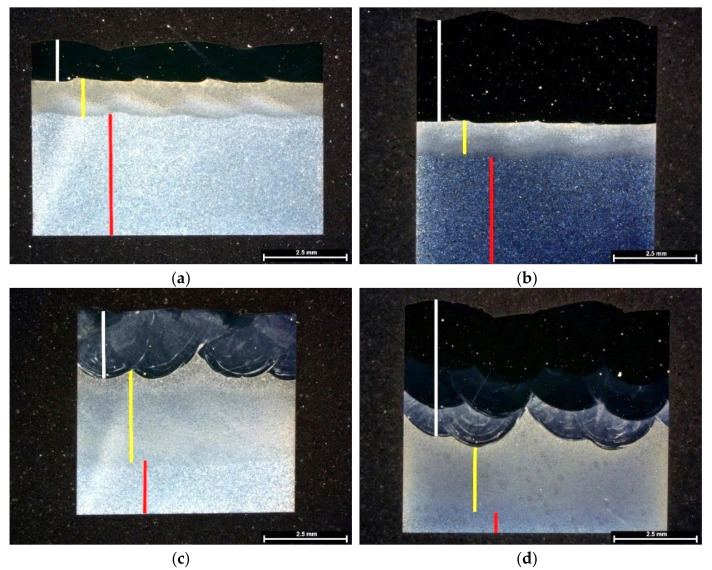
The dark field images obtained by OM technique showing cross-sections of deposited 18Ni300 alloy: (**a**) one layer of deposition and (**b**) three layers of deposition on a non-preheated substrate, (**c**,**d**) similar depositions on the preheated substrate, white insets show deposited layer(s), yellow insets signify HAZ, and red ones represent substrate.

Metallography in the current study involves OM observations from etched cross-sections. Figure 2 reveals the morphology of deposited materials consisting, from top to the bottom, on deposited layer(s), heat-affected zone (HAZ) and the substrate material, illustrated by white, yellow and red insets, respectively. Moreover, cracking defects were not observed; however, some pores were seen, independent of processing conditions, having a maximum diameter of 60 to 70 µm.

This process involves heating the substrate material in some depth forming the heat-affected zone (HAZ) since laser deposition involves melting metal powders with a portion of substrate material, so-called dilution. Figure 2 shows the presence of HAZ in the materials processed in this study. Regarding one layer deposition without preheating (Figure 2a), the HAZ is sharp with a curvy-like morphology at the ending zone in the substrate and a thickness of 1.1 mm ± 0.1 mm. This layer is similar in the three-layered deposition, Figure 2b, that is, 1.0 mm ± 0.1 mm thick, confirming that the HAZ depth is independent of the number of depositions. Regarding the effect of preheating on the extent of HAZ and its morphology, it enlarged the HAZ with a smooth ending zone in the substrate, Figure 2c,d. In the one-layer deposition produced with preheating substrate (Figure 2c), it appears that the HAZ is divided into two regions: a smaller layer consists of large grains beneath the deposition layer followed by a thin microstructure. The HAZ depth of this deposition is 2.7 mm ± 0.3 mm thick; this measurement is 2.5 mm ± 0.4 mm for the preheated three-layered deposition. Moreover, it seems that the HAZ formed by three depositions is more uniform than that of one deposition. Therefore, the depth of HAZ is independent of the number of deposition layers.

Figure 2a,b reveals that a tiny dilution zone formed at the bonding face with substrate is independent of the number of deposition layers, whereas this depth changed for the preheated substrates (Figure 2c,d, i.e., the deposition morphology on preheated substrates consists of deeper dilution curves). 

Besides, Figure 2 illustrates the presence of a brighter deposition layer mainly the first layer deposited on preheated substrates; this difference, in contrast, is attributed to the dilution effect provoked by preheating and it is almost eliminated in the last layer of the deposition on the preheated material (Figure 2d). 

### 3.2. Evolution of Chemical Composition across Depositions Affected by Processing Conditions

**Figure 3:**
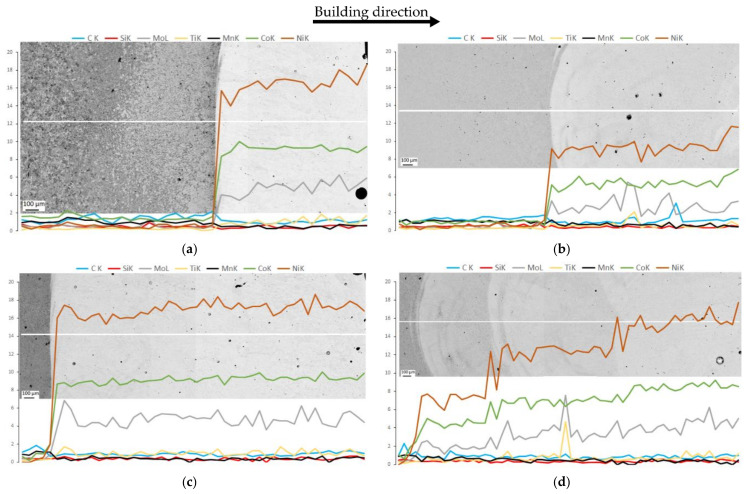
The EDS profiles (in wt.%) across the HAZ to deposited layer(s): (**a**,**b**) one deposition produced without and with preheating, respectively, (**c**,**d**) three depositions, produced similarly.

Figure 3 presents linear chemical compositions across the same metallography specimens illustrated in Figure 2. This analysis justifies the contrast differences observed in the dark field OM images that appeared in Figure 2. The presence of a less dark deposition layer, comparing images in Figure 2a,c, is attributed to the decrease in the concentration of alloying elements in the one-layered deposition received preheating, mainly in Ni, Co and Mo, Figure 3a,b. Similarly, the difference in contrast observed in the first and second layer of the three-layered deposition with preheating, Figure 2d, caused by dilution effect, i.e., the strongest occurred in the first layer, and gradually decreased in consecutive layers, is almost eliminated in the last deposited layer, Figure 3d. Thus, the most uniform deposition, taking into account chemical composition, was obtained in the three depositions produced without preheating.

Thus, strong metallurgical bonding between 18Ni300 alloy and AISI 1045 steel occurred for the depositions that received preheating condition. Moreover, this processing condition influenced the extent of HAZ in the substrate material.

### 3.3. Effect of Number of Layers and Preheating on the Microstructure of HAZ

The substrate material beneath the deposited layer receives heat from the molten pool formed during deposition. In the meantime, the substrate material functions as a cooling media, consequently, microstructure changes in comparison with that of the virgin substrate, forming HAZ. Recent studies showed that the HAZ properties depended on the temperature of the substrate, in the room temperature or heated up to 250 °C [26,27,28]. The substrate used in the current study is AISI 1045 that is susceptible to martensitic transformation, depending on the cooling rate [25]. Thus, substrate preheating was implemented, at a temperature above the Ms temperature, which is 400 °C, to decrease the cooling rate in the area beneath the deposition layer, in the substrate. This methodology was applied to avoid martensite transformation. 

**Figure 4:**
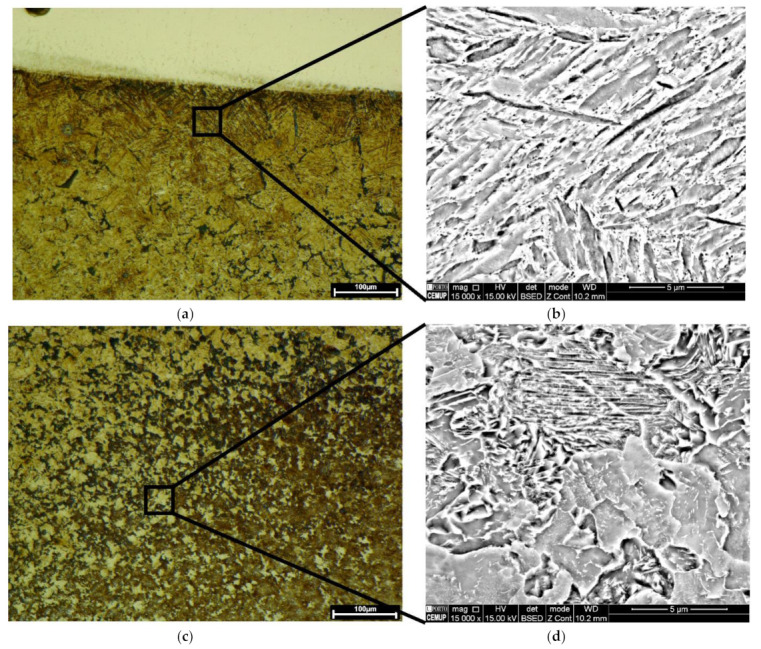
The HAZ microstructure in the one-layered deposition produced without preheating: OM and SEM images, respectively, of (**a**,**b**) martensite exists beneath the deposited layer, and (**c**,**d**) the mixture of bainite and pearlite at the end of HAZ (SEM images were acquired from black insets).

Figure 4 illustrates the microstructure across the HAZ of the one-layered deposition produced without preheating. These observations confirm the presence of lath martensite at the topmost region of the HAZ, Figure 4a,b. This microstructure gradually transforms to the mixture of bainite, most likely, and pearlite reaching the end region of HAZ in the substrate, Figure 4c,d. 

**Figure 5:**
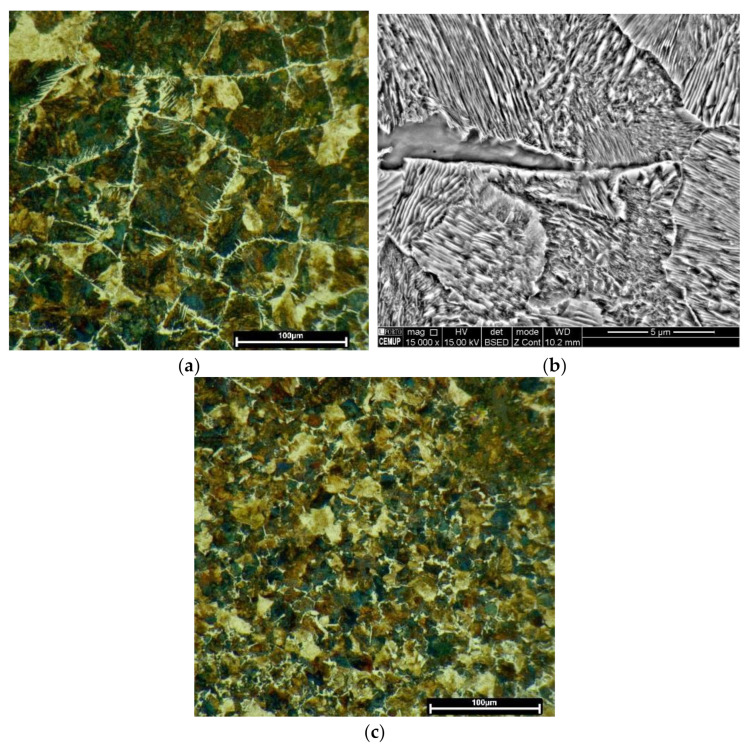
The HAZ microstructure formed in the one-layered deposition produced with preheating: OM and SEM images, respectively, of (**a**,**b**) the bainite beneath the deposited layer, and (**c**) fine-grained structure at the end of HAZ.

Regarding the effect of preheating on the one-layered deposition, the martensitic transformation did not happen, as evolved at the topmost region of the HAZ in Figure 4a. The HAZ microstructure transformed into bainite, most likely, and Widmanstatten ferrites, formed at primary austenite grain boundaries, Figure 5a,b. Fine pearlite and ferrite grains dominated the microstructure, reaching the end region of HAZ, Figure 5c.

**Figure 6:**
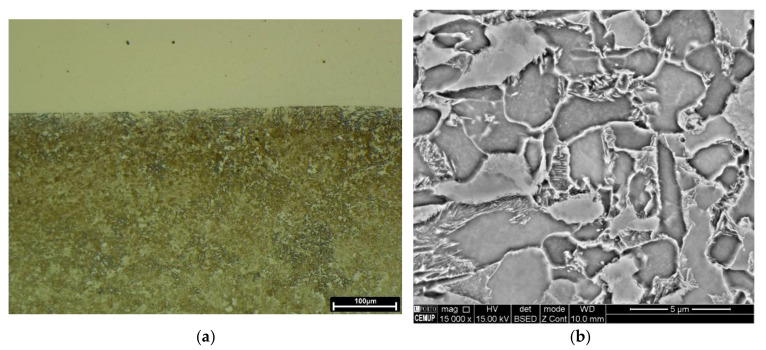

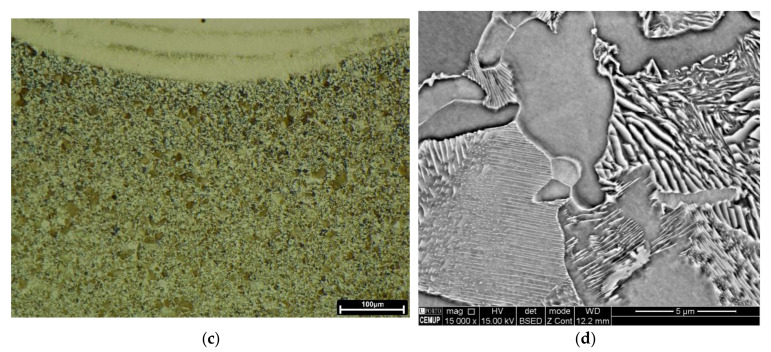
The microstructure of HAZ formed in the three-layered deposition, respectively OM and SEM images: (**a**,**b**) produced without preheating; (**c**,**d**) with preheating.

Figure 2 revealed that the extent of HAZ did not change by the deposition of three layers. However, Figure 6a,b reveals that the HAZ microstructure changed in comparison with that of the one-layered deposition, seen in Figure 4. Figure 6a,b confirms that the microstructure across the HAZ region of the three-layered deposition produced without preheating, from the topmost region to the end of HAZ, is composed of bainite, likely, fine pearlite, and ferrite. Whereas, fine structured pearlite grains and ferrite constitute the HAZ of the three-layered deposition produced by preheating, Figure 6c,d. The microstructure turns into fine grains by reaching the end of HAZ.

Thus, the HAZ microstructure formed in the AISI 1045 substrate suffered martensitic transformation during direct energy deposition of maraging 18Ni300 alloy. This microstructure was obtained in the substrate that was not preheated, caused by a fast cooling. Martensite did not remain in the HAZ of the three-layered deposition without preheating, this effect can be attributed to a tempering effect caused by further depositions. This diffusion-less transformation was eliminated when the substrate was preheated, indicating the efficiency of preheating on cooling rate.

### 3.4. Interfaces of Deposited 18Ni300 Powder Alloy and AISI 1045 Substrate

#### 3.4.1. Microstructure of Interface Affected by Processing Conditions

**Figure 7:**
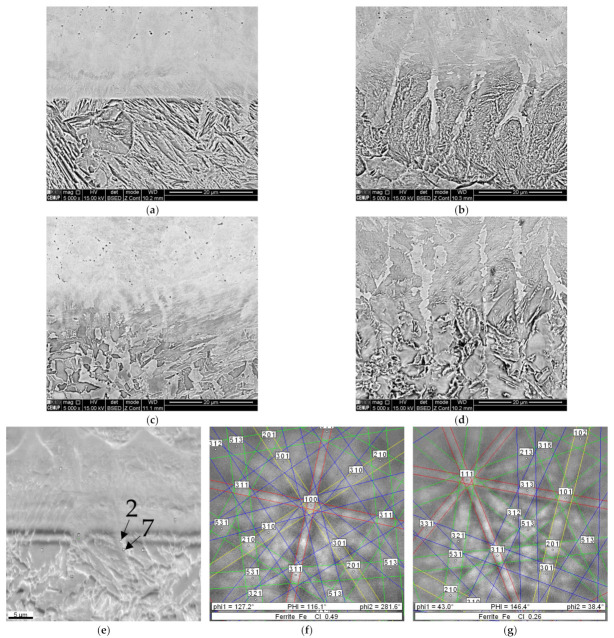
The SEM/BSE images of interfaces from (**a**,**b**) one-layered deposition produced without and with preheating, respectively, (**c**,**d**) three-layered depositions without and with preheating; (**e**) the SEM/SE image from the same interface shown in the image (**c**) showing some points for performing Kikuchi indexation analysis, (**f**,**g**) are Kikuchi patterns obtained from zones 2 and 7 indicated by black arrows in the image, showing the directions of the crystals (**e**).

Figure 7 confirms that the interfaces of the deposition layers produced in the current study consist of epitaxial growth, independent of prehearing condition. This bonding phenomenon mainly occurred for the ferritic phase, as confirmed by Kikuchi pattern indexation analyses, Figure 7e–g. However, the width of the epitaxial bands was affected by the excess of heat received from preheating process or successive depositions, thick route-like zones observed in Figure 7b,d, which leads to the increase in diffusion and crystal growth at these preferential zones.

#### 3.4.2. Chemical Composition of Interfaces Affected by Processing Conditions

**Figure 8:**
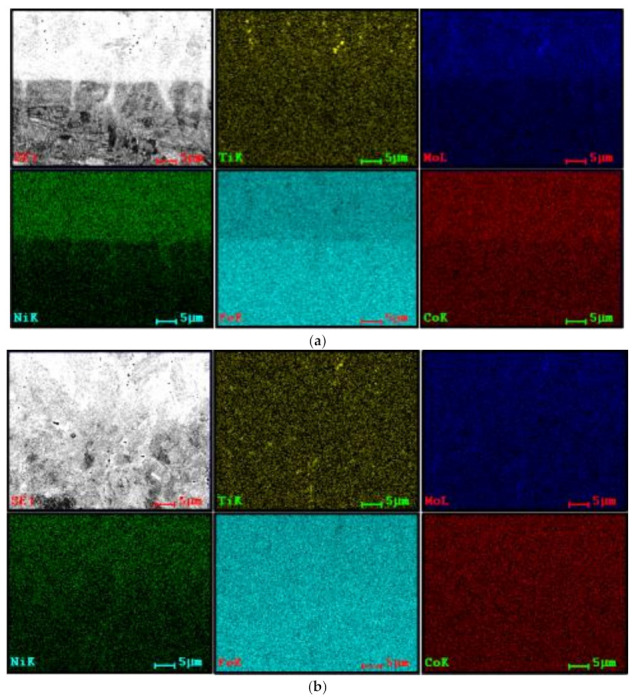
The SEM and EDS mapping analyses performed at the interface of three-layered depositions produced: (**a**) without and (**b**) with preheating process.

Nevertheless, Figure 8 illustrates that the chemical composition at the interfaces of three-layered depositions is almost homogeneous, the preheating or further deposition layers preserved the homogeneity; however, a transition line is distinguished clearly at the interface of the material produced without preheating, which is very sharp for the Ni, Figure 8a. These EDS analyses reveal the presence of some titanium compound particles, yellow-coloured spots, with the possibility of being complex oxides or carbides, as confirmed by local EDS analysis. Some researchers reported the formation of titanium nitride [29].

#### 3.4.3. Grains Evolutions in the Interfaces Affected by Processing Conditions

**Figure 9:**
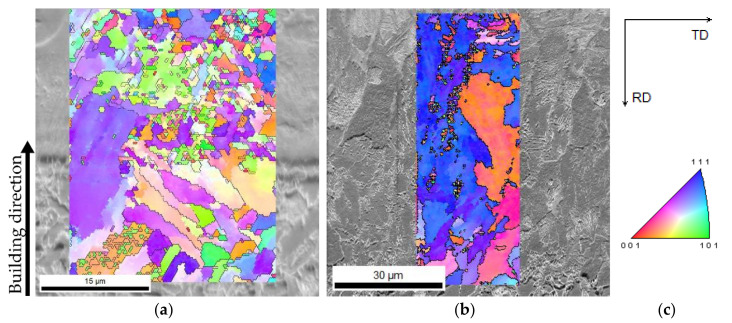
Interface crystallographic information of the three-layered depositions: SEM image superimposed by IPF analysis, the black arrow shows the building direction from the substrate to the deposited layer, of the material produced (**a**) without and (**b**) with preheating; (**c**) the direct and index maps of the analysing surfaces by EBSD method.

The analysis of interface is noteworthy since this zone constitutes dissimilar materials. Regarding the crystallographic information and grain structure at these interfaces, as seen in Figure 9, the interfaces are composed of large to fine grains, mainly at the interface produced without preheating, Figure 9a. The preheating influenced the interface evolving large and elongated/columnar single grain from the substrate to the diluted zone, Figure 9b, as expected from highlighted epitaxial growth seen in Figure 7d. This morphology formation is consistent with similar studies [30]. Moreover, the IPF analyses reveal a preferential orientation to (111) direction, occurred in the interface that received preheating condition, as seen in Figure 9b. Regarding the acquisition of EBSD analysis for the material produced by preheating condition, since the transition zone is not as thin as the deposition without preheating, Figure 9a, scanning was performed in a larger area, maintained the step size.

### 3.5. Depositions of 18Ni300 Alloy Powder Produced by DED

#### 3.5.1. Evolution of Microstructure and Chemical Composition across Depositions

**Figure 10:**
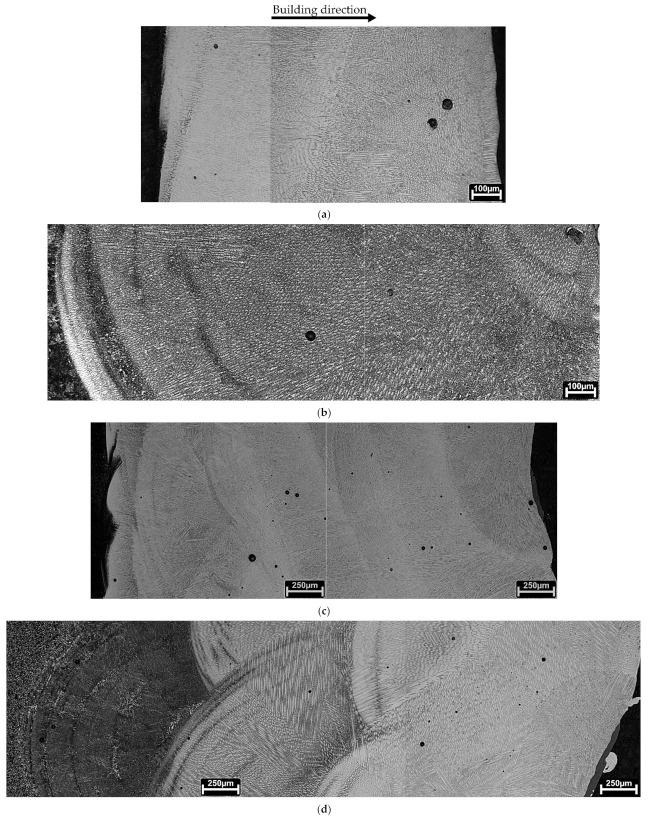
OM images from the etched cross-sections of (**a**,**b**) one deposited layer produced without and with preheating, respectively, (**c**,**d**) three layers deposition, produced similarly.

Deposition process by DED methodology involves melting metal powders on the substrate surface, consequently it can include the melting of the substrate forming dilution, followed by the transformation of liquid to solid. This solidification is fast and similar to welding processes; however, the morphology of microstructure (planar, cellular, columnar dendrites or equiaxed type) is influenced by temperature gradient and growth rate at the solidifying front [31]. The microstructure of deposited materials is mainly composed of dendrites influenced by the heat dissipation during the solidification process [22] as well as the heat transfer from neighbouring lines, which means overlapping, the variation of microstructure depends on the distance from the substrate and of the local cooling rate [32]. Regarding the one-layered deposition, as seen in Figure 10a, the microstructure is almost composed of columnar dendrites close to the substrate, uniformly orientated with the building direction; however, this morphology changes to cellular/equiaxial morphology reaching to the topmost region. The formation of columnar dendrites is attributed to the decrease in the cooling rate, caused by the room temperature substrate; the transformation of columnar to equiaxial form caused by the change in the ratio of temperature gradient and solidification rate reaching to the topmost region of the deposited layer [32,33]. Figure 10b illustrates the formation of cellular/equiaxial dendrites in the one-layered deposition with preheating. This morphology seems to be dominant in this deposition, it is even seen from the bottom region of the diluted zone, that means, the excess of heat through preheating could contribute to the favour of cellular/equiaxial dendrites. 

Regarding the microstructure of three-layered depositions, Figure 10c,d, elongated and cellular/equiaxial dendrites constituted the whole of the depositions, independent of preheating condition. However, the growth direction does not seem perpendicular to the substrate, which means, it has been affected by the heat transfer from neighbouring lines. Dendrites are less elongated reaching the topmost region. These results are consistent with a similar study [22] in which the authors attributed the decrease in dendrite size to overlapping. Figure 10c,d highlights the difference in the thickness of dendrites, i.e., deposited material produced on a preheated substrate is composed of thicker dendrites, Figure 10d, than the deposition without preheating, the accumulation of heat was in the favour of interspace growth from almost 6 microns to 12 microns, respectively, in without and with preheating depositions. These values are not as big as the size of intercellular spacing reported by other authors due to the processing conditions such as the energy of deposition, overlapping, etc. [29,34]. Regarding the microstructure illustrated in Figure 10d, it is composed of three distinct layers as seen in Figure 2d, the microstructure presents three different contrasts influenced by the dilution effect and the variation of concentrations in the chemical composition of the consecutive layers, as presented by linear analysis in Figure 3d.

**Figure 11:**
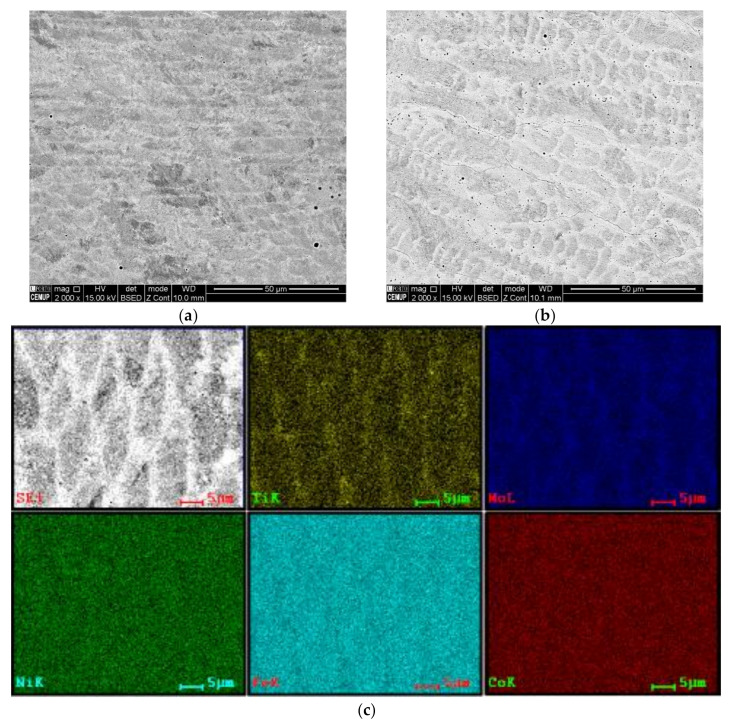

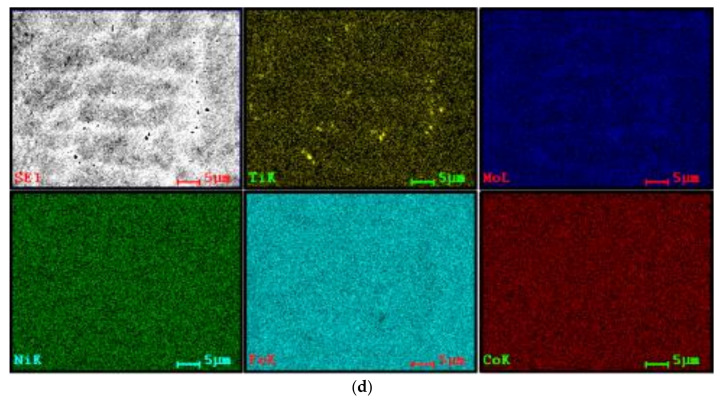
The SEM images acquired from almost similarly positioned regions of three-layered depositions: (**a**) without and (**b**) with preheating process; (**c**,**d**) are EDS mapping analyses obtained from respective (**a**,**b**) microstructures.

The maraging 18Ni300 alloy processed by the DED process usually demonstrates a cellular microstructure consisting of packets of lath martensite [29]. The martensitic transformation from the primary austenite phase occurs by a shear deformation during the cooling stage. The microstructure of the deposited materials in the current study shows dendritic-cellular morphologies, Figure 10c,d and Figure 11a,b, these observations reveal that preheating has affected the interspacing in cells embedding martensite laths. That is consistent with similar studies [34].

Figure 11c,d illustrates the elemental distribution of two similar zones, taking into account the morphology of dendrites, cellular/equiaxed, of the three-layered depositions produced without and with preheating preparations. The chemical composition is homogeneous; however, a slight concentration of Ti is observed in the interspace dendrites in the material produced without preheating, whereas it is not observed for the preheated material. The performance of local EDS analysis confirms that some particles represent complex Ti, Mo compounds.

#### 3.5.2. Crystallographic Information of 18Ni300 Depositions Produced by DED

**Figure 12:**
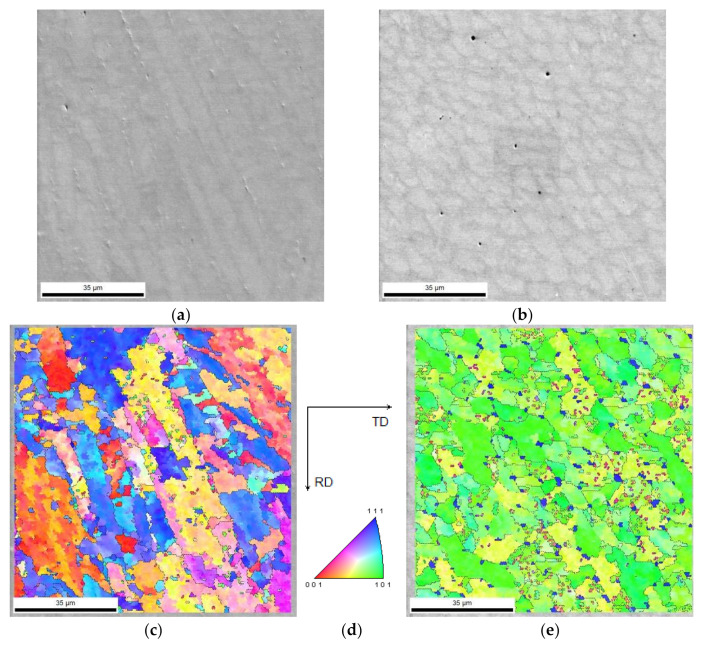

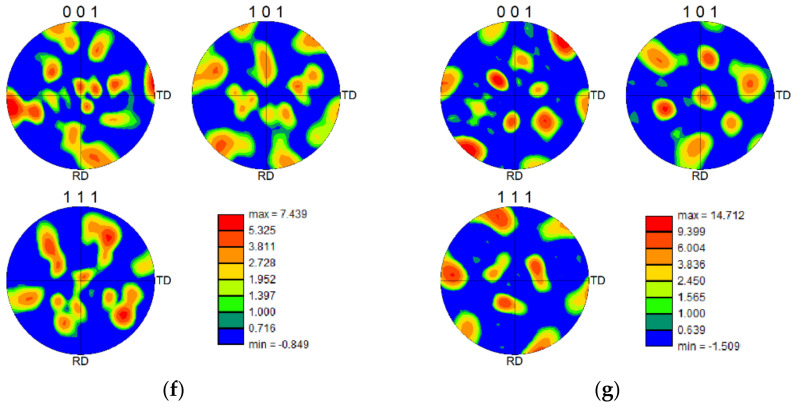
Microstructure and crystallographic information of the deposited alloy without preheating: (**a**,**b**) the SEM images from two distinct regions almost 500 µm respectively far from the interface with substrate and the topmost external surface, (**c**–**e**) IPF and corresponding indexation map of the similar zones, (**f**,**g**) texture analysis of the two zones (obtained by EBSD analysis).

**Figure 13:**
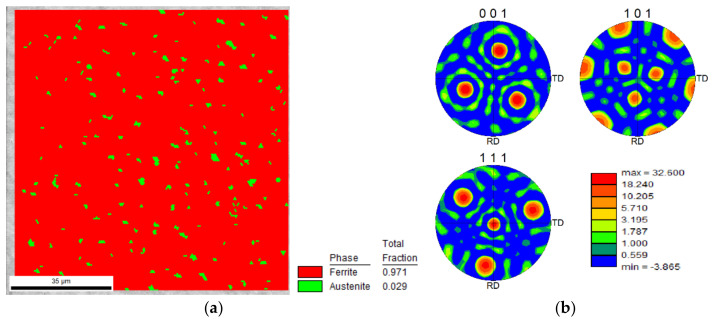
The EBSD analyses results show (**a**) phase distribution map and (**b**) pole figure texture map for the three-layered deposition produced without preheating.

The crystallographic structure of the 18Ni300 alloy depositions, across the three-layered depositions was studied by EBSD analysis. Regarding the deposition produced without preheating, Figure 12a,b illustrates the microstructure morphology of the two zones, constituted by elongated/columnar and equiaxed dendrites respectively near the interface and the topmost region of the third deposited layer. Corresponding IPF images shown in Figure 12c–e reveal that the microstructure, which is in the normal direction, is composed of grains with a slight preferential orientation with (111) and (001) for the elongated dendritic structure and a strong preferential orientation with (101) for the equiaxed dendritic zone (Figure 12c–e). These distributions provoked a texture for the cellular microstructure, confirmed by the analysis illustrated in Figure 12g. Three pole figures illustrate a strong texture, in particular, the (101) pole figure has a peak perpendicular to the observing surface of the specimen. The formation of this strong texture can be attributed to the heat dissipation that occurred unidirectionally. Besides, in Figure 12e, small blue spots are seen representing a preferential crystallography orientation, the IPF image shows that all these spots have the (111) direction perpendicular to the sample plane. These blue spots are most probably austenite, confirmed by the EBSD phase map shown in Figure 13a, showing a strong texture, Figure 13b, with one strong peak observed at the centre of the pole figure for (111) direction, which is consistent with the blue colour observed on the IPF map of Figure 12e.

Regarding the presence of the austenite phase seen in Figure 13b, the heat accumulated from previous deposition layers could provoke the reversion of the small proportion of matrix, martensite, to austenite that occurs as the most common phenomenon in the aging treatment during the cooling process [29]. 

**Figure 14:**
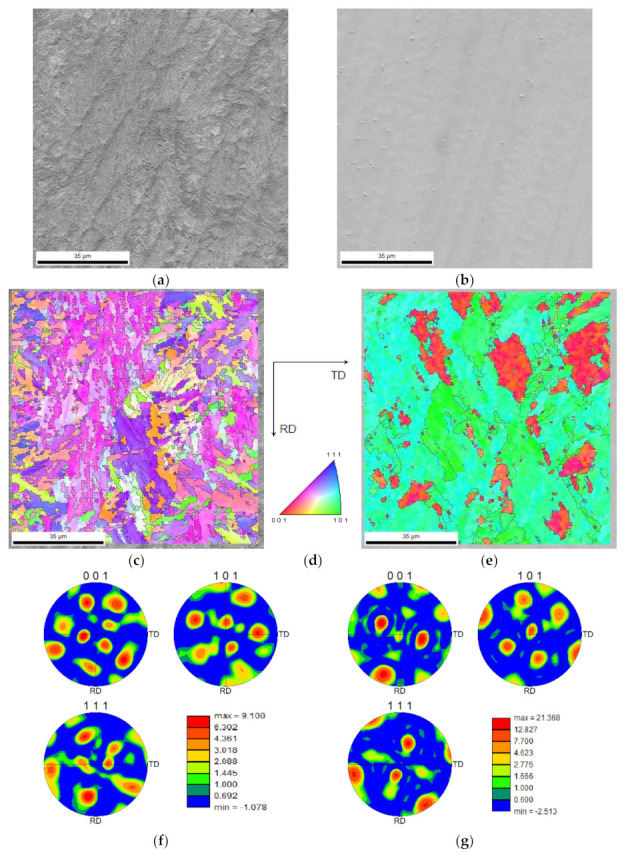
Microstructure and crystallographic information of the deposited alloy produced by applying preheating condition: (**a**,**b**) the SEM images from two distinct regions, in the centre of the first layer and that of the third layer, respectively, (**c**–**e**) IPF and corresponding indexation map of the similar zones, (**f**,**g**) texture analysis of the two zones (obtained by EBSD analysis).

The crystallographic analyses of the three-layered deposition, produced with preheating, were performed in two distinct distances from the substrate. One, in the middle of the first deposited layer, Figure 14a, the other in the middle of the third layer as shown in Figure 14b. Both zones are constituted by almost columnar dendrites; however, the first microstructure presents finer grains than that of the topmost region, Figure 14c–e. The area at the topmost region shows a strong preferential crystallographic orientation led to a strong texture to (101) direction, Figure 14f,g.

**Figure 15:**
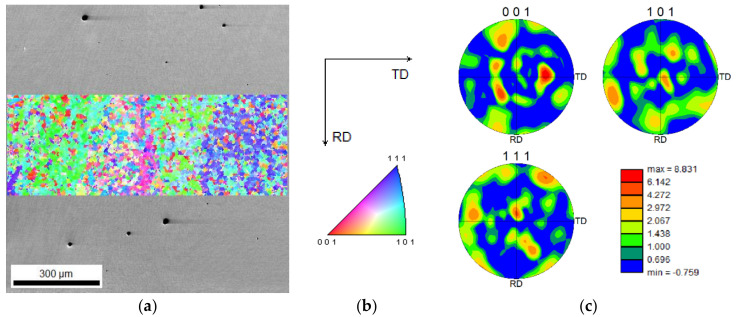
(**a**) The IPF inset superimposed on a SEM image in the middle of the third layer of the three-layered deposition (**b**) accompanied with the corresponding index map, and (**c**) the texture analysis.

Figure 15 reveals that the presence of strong microtextured structure, as shown in Figure 12g or Figure 14g, reduces at larger areas. However, a weak preferential orientation is observed along the building direction, Figure 15a.

### 3.6. Hardness Distribution from HAZ to Deposition

**Figure 16:**
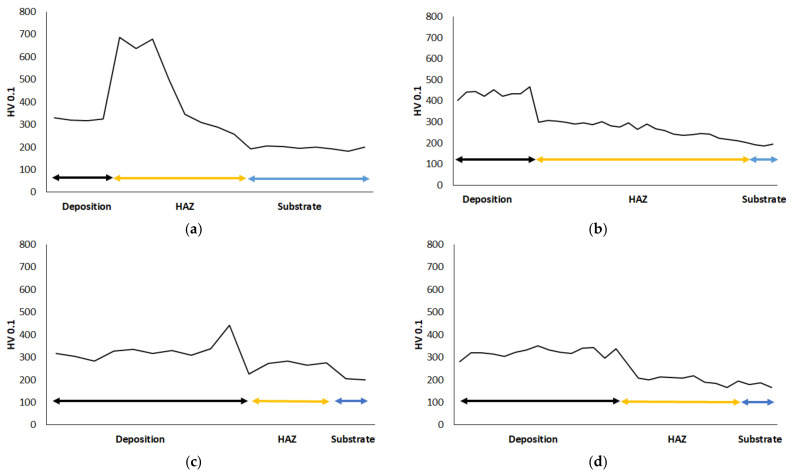
Microhardness graphs, measurements from the deposited layers to the substrate: (**a**) one deposition without preheating, (**b**) one deposition with preheating, (**c**) three-layered deposition produced without preheating, and (**d**) three-layered-deposition with preheating.

Figure 16 illustrates the hardness evolution across the deposited maraging 18Ni300 alloy depositions as well as that of the HAZ regions and the AISI 1045 substrates. One-layered deposition on a substrate without preheating has a 323 ± 15 HV0.1 hardness, reaching the HAZ, seen in Figure 16a, the hardness dramatically increases due to the martensitic transformation, as seen in Figure 4. Then, the hardness decreases due to the formation of bainite and fine pearlite. The one-layered deposition alloy received preheating condition shows a larger hardness, 438 ± 28 HV0.1 this increase in hardness is attributed to the dilution effect presented in Figure 3b. As seen in Figure 16b, the hardness decreases reaching the HAZ following a smooth transition to the substrate. The hardness of the three-layered deposition without preheating is 331 ± 41 HV0.1, that is, similar to one layer; however, as seen in Figure 16c, the transition to the HAZ is smooth having a hardness similar to that of the substrate material. Although the chemical composition across the three-layered deposition alloy with the preheating condition faced an incremental trend in the main alloying elements, from the substrate to the topmost region of the deposition, seen in Figure 3d, the hardness across this deposition is 322 ± 19 HV0.1. Moreover, Figure 16d presents that this deposition shows a similar behaviour as observed in the material without preheating. 

Therefore, the performance of successive depositions smoothened the hardness evolutions, the hardness increased by strong dilutions in preheated single-layered depositions. These hardness values obtained in the current study are similar to other studies [34]; however, processing conditions and substrate can lead to differences. Successive deposition production, performed in the current study, acted as tempering HAZ, which can be achieved by the application of preheating as well. This effect can eliminate the stress concentrations caused by large and sudden hardness variations. 

## 4. Conclusions

The deposition of maraging 18Ni300 alloy on AISI 1045 steel was successfully performed by direct energy deposition process, taking into account the absence of cracks. In the current study, four conditions were evaluated: two one-layered depositions respectively without and with preheating substrate, similarly, two other depositions consisted of three layers. Microscopy observations of the crossed sections revealed that the dilution, as well as the size of the heat-affected zone, was significantly increased by the performance of preheating the substrate sheet, independently of the number of deposited layers. The chemical composition of the deposition layers without preheating was steady. The preheating led to the elimination of martensite transformation in the HAZ, a similar result was achieved in successive depositions. The latter processing strategy influenced the microstructure as a tempering treatment, evolving a smooth transition in hardness from interface to the substrate. Metallurgical bonding at the obtained interfaces is strong, confirmed by epitaxial growth or the formation of elongated grains, affected by preheating conditions. The chemical composition from the interface to the topmost of the deposited layers, produced in the current study, is uniform; however, few Ti complex compounds have formed. The dendrite morphology was influenced by the preheating condition, i.e., cellular/equiaxed dendrites dominated the one-layered deposition with preheating. Moreover, preheating led to increase the interspace growth in dendrites. It was clearly observed that the heat propagation and/or dissipation, affected by successive deposition across the layers, influenced the direction of dendrites growth. The electron backscatter diffraction (EBSD) evaluations highlighted the presence of an almost strong texture in the cellular/equiaxed zones formed in the microstructure of depositions independent of preheating condition. Although EBSD analysis revealed microtextured structures at different positions in the deposited planes, textured structure was not very strong for larger areas.
